# The cytological mechanism of the peach haploid producing triploid offspring

**DOI:** 10.1093/hr/uhae316

**Published:** 2024-11-18

**Authors:** Xin Liu, Dandan Li, Yu Zhang, Xin Zhou, Shangde Wang, Jianbo Zhao, Jiying Guo, Quan Jiang, Fei Ren

**Affiliations:** Institute of Forestry and Pomology, Beijing Academy of Agriculture and Forestry Sciences, Haidian District, Beijing 100093, China; Key Laboratory of Biology and Genetic Improvement of Horticultural Crops (North China), Ministry of Agriculture and Rural Affairs, Haidian District, Beijing 100093, China; Beijing Engineering Research Center for Deciduous Fruit Trees, Haidian District, Beijing 100093, China; Key Laboratory of Urban Agriculture (North China), Ministry of Agriculture and Rural Affairs, Haidian District, Beijing 100093, China e Department of Pomology, College of Horticulture, China Agriculture University, Haidian District, Beijing 100193, China; Institute of Forestry and Pomology, Beijing Academy of Agriculture and Forestry Sciences, Haidian District, Beijing 100093, China; Key Laboratory of Biology and Genetic Improvement of Horticultural Crops (North China), Ministry of Agriculture and Rural Affairs, Haidian District, Beijing 100093, China; Beijing Engineering Research Center for Deciduous Fruit Trees, Haidian District, Beijing 100093, China; Key Laboratory of Urban Agriculture (North China), Ministry of Agriculture and Rural Affairs, Haidian District, Beijing 100093, China e Department of Pomology, College of Horticulture, China Agriculture University, Haidian District, Beijing 100193, China; Institute of Forestry and Pomology, Beijing Academy of Agriculture and Forestry Sciences, Haidian District, Beijing 100093, China; Key Laboratory of Biology and Genetic Improvement of Horticultural Crops (North China), Ministry of Agriculture and Rural Affairs, Haidian District, Beijing 100093, China; Beijing Engineering Research Center for Deciduous Fruit Trees, Haidian District, Beijing 100093, China; Key Laboratory of Urban Agriculture (North China), Ministry of Agriculture and Rural Affairs, Haidian District, Beijing 100093, China e Department of Pomology, College of Horticulture, China Agriculture University, Haidian District, Beijing 100193, China; Institute of Forestry and Pomology, Beijing Academy of Agriculture and Forestry Sciences, Haidian District, Beijing 100093, China; Key Laboratory of Biology and Genetic Improvement of Horticultural Crops (North China), Ministry of Agriculture and Rural Affairs, Haidian District, Beijing 100093, China; Beijing Engineering Research Center for Deciduous Fruit Trees, Haidian District, Beijing 100093, China; Key Laboratory of Urban Agriculture (North China), Ministry of Agriculture and Rural Affairs, Haidian District, Beijing 100093, China e Department of Pomology, College of Horticulture, China Agriculture University, Haidian District, Beijing 100193, China; Institute of Forestry and Pomology, Beijing Academy of Agriculture and Forestry Sciences, Haidian District, Beijing 100093, China; Key Laboratory of Biology and Genetic Improvement of Horticultural Crops (North China), Ministry of Agriculture and Rural Affairs, Haidian District, Beijing 100093, China; Beijing Engineering Research Center for Deciduous Fruit Trees, Haidian District, Beijing 100093, China; Key Laboratory of Urban Agriculture (North China), Ministry of Agriculture and Rural Affairs, Haidian District, Beijing 100093, China e Department of Pomology, College of Horticulture, China Agriculture University, Haidian District, Beijing 100193, China; Institute of Forestry and Pomology, Beijing Academy of Agriculture and Forestry Sciences, Haidian District, Beijing 100093, China; Key Laboratory of Biology and Genetic Improvement of Horticultural Crops (North China), Ministry of Agriculture and Rural Affairs, Haidian District, Beijing 100093, China; Beijing Engineering Research Center for Deciduous Fruit Trees, Haidian District, Beijing 100093, China; Key Laboratory of Urban Agriculture (North China), Ministry of Agriculture and Rural Affairs, Haidian District, Beijing 100093, China e Department of Pomology, College of Horticulture, China Agriculture University, Haidian District, Beijing 100193, China; Institute of Forestry and Pomology, Beijing Academy of Agriculture and Forestry Sciences, Haidian District, Beijing 100093, China; Key Laboratory of Biology and Genetic Improvement of Horticultural Crops (North China), Ministry of Agriculture and Rural Affairs, Haidian District, Beijing 100093, China; Beijing Engineering Research Center for Deciduous Fruit Trees, Haidian District, Beijing 100093, China; Key Laboratory of Urban Agriculture (North China), Ministry of Agriculture and Rural Affairs, Haidian District, Beijing 100093, China e Department of Pomology, College of Horticulture, China Agriculture University, Haidian District, Beijing 100193, China; Institute of Forestry and Pomology, Beijing Academy of Agriculture and Forestry Sciences, Haidian District, Beijing 100093, China; Key Laboratory of Biology and Genetic Improvement of Horticultural Crops (North China), Ministry of Agriculture and Rural Affairs, Haidian District, Beijing 100093, China; Beijing Engineering Research Center for Deciduous Fruit Trees, Haidian District, Beijing 100093, China; Key Laboratory of Urban Agriculture (North China), Ministry of Agriculture and Rural Affairs, Haidian District, Beijing 100093, China e Department of Pomology, College of Horticulture, China Agriculture University, Haidian District, Beijing 100193, China; Institute of Forestry and Pomology, Beijing Academy of Agriculture and Forestry Sciences, Haidian District, Beijing 100093, China; Key Laboratory of Biology and Genetic Improvement of Horticultural Crops (North China), Ministry of Agriculture and Rural Affairs, Haidian District, Beijing 100093, China; Beijing Engineering Research Center for Deciduous Fruit Trees, Haidian District, Beijing 100093, China; Key Laboratory of Urban Agriculture (North China), Ministry of Agriculture and Rural Affairs, Haidian District, Beijing 100093, China e Department of Pomology, College of Horticulture, China Agriculture University, Haidian District, Beijing 100193, China

## Abstract

Peach is one of the most economically valuable fruit trees. Haploid peach trees occur spontaneously at very low frequencies and they are usually highly sterile. Therefore, the haploid with partial fertility is an extremely rare germplasm, which is highly valuable to genetic research and breeding programs. In this study, we investigated the cytological mechanism underlying the fertility of a peach haploid mutant ‘9-D’ derived from a spontaneous mutation. Cytologic evaluation and flow cytometry analysis demonstrated that ‘9-D’ is a pure haploid. Scanning electron microscope analysis revealed a considerable proportion of abnormal pollen grains in ‘9-D’. Pollen viability assay by Alexander staining showed that 50.4% of pollen grains from ‘9-D’ were viable. However, the pollen germination assay showed that only 7.6% of the pollen grains could germinate normally. Investigation of the chromosomal behavior of pollen mother cells at different stages of meiosis showed that pollen mother cells of ‘9-D’ lacked the process between anaphase I and prophase II of meiosis. Various types of sporophyte morphology were observed in haploid pollen mother cells at the tetrad stage. Measurement of the diameter of pollen grains indicated the presence of pollen with 2*x* ploidy in ‘9-D’. The offspring of ‘9-D’ were predominantly triploid or triploid aneuploid. The triploid offspring were more likely derived from the 2*x* male gametophyte combined with the haploid female gametophyte, which may explain the reason why ‘9-D’ has fertility. This study not only expands our understanding of haploid fertility mechanisms, but is also useful for ploid breeding programs in peach.

## Introduction

Peach (*Prunus persica* (L.) Batsch) belongs to the Rosaceae family and is the third largest deciduous fruit tree species in China. Peaches are staple fruit that are available throughout the year. China ranks first globally in terms of peach cultivation area and yield. In recent years, peach breeding has made important progress in China, and peach has become the tree species with the highest market share of China’s independent varieties among major fruit tree species [1, 2]. Breeding new varieties is crucial for the sustainable development of the peach industry, and ploidy breeding is one of the important techniques for creating breeding innovation. Peach is a diploid species (2*n* = 2*x* = 16, *x* means basic number of chromosomes), and most cultivated peach varieties and their wild relatives are diploid [3]. A number of germplasms with different ploidy levels have been developed by artificial induction or hybridization. Among all the ploidy germplasm of peach, haploid is the most abundant, and has been reported in >30 literature [4, 5]. These peach ploid germplasm originated from natural pollination or artificial hybridization [5, 6]. There are great differences in genetic, physiological, and biochemical aspects between diploid and haploid peaches. Haploid plants are extremely weak in growth with narrow and light green-colored leaves, short internodes, small flowers, miniature fruits, and low fertility compared to diploid plants [7, 8]. Since haploid plants contain only a single set of chromosomes, they are valuable germplasm resources for genetic study and breeding innovation [9]. Understanding of mechanisms behind peach haploid formation can provide theoretical support for the artificial induction of haploid plants and lay the foundation for haploid breeding.

The majority of plant haploids have empty pollen grains and are thus sterile [10]. Studies on the meiosis process in pollen grains of haploid plants, such as hot pepper, lily, *Brassica rapa*, and eggplant, show that unbalanced gametes are generated when microsporocytes have univalent and multivalent in the prophase stage of meiosis, chromosome bridges, and chromosome fragments in the anaphase stage of meiosis, and unequal division or multipolar division in the telophase stage [11–14]. Thus, microsporocytes cannot develop normally, leading to sterile gametes and reduced pollen fertility. Linkage and crossing over cannot occur in haploids, and univalent separate unequally at the first division, resulting in microsporocytes with unequal DNA distribution. However, *MiMe* mutant can turn meiosis into mitosis, thereby abolishing recombination and generating haploid gametes [15]. Several studies have shown that haploid offspring can be triploid, diploid, or aneuploid in peach [3]. Thus, the reason why plant haploids exhibit a high degree of sterility could be attributed to their inability to produce balanced gametes because of abnormal meiosis, and the degree of sterility varies among plant species.

Natural doubling of gametic chromosomes has been reported in many species, most of which are related to unreduced gametes. Unreduced gametes usually refer to those with somatic chromosome numbers that are formed as a result of abnormal development of megaspore or microspore cells during meiosis or mitosis due to genetic or environmental factors [16]. The formation of natural doubling gametes is an important pathway for the production of polyploid plants and a driver of polyploid formation in nature [17, 18]. It occurs universally in the plant kingdom, and almost all plants can form unreduced gametes [19]. To date, the formation of unreduced gametes has been detected in at least 85 genera [20]. Unreduced gametes have been reported in most fruit tree species such as apple, pear, grape, strawberry, and *Citrus* [21]. Premeiotic genome doubling, first division restitution, second division restitution, postmeiotic genome doubling, and abnormal cytoplasmic division are important factors required for unreduced pollen formation in plants [22, 23]. During microsporogenesis, meiosis I or meiosis II chromosome segregation that is not accompanied by cytoplasmic division or abnormal cytoplasmic division can lead to unreduced pollen production [24]. Mutations such as meiotic delay, meiosis II deletion, and second cytoplasmic division failure are the main reasons for the formation of unreduced female gametes [25]. Unreduced gametes can be identified by pollen grain diameter determination, pollen DNA content determination, cytological characterization, and hybridization [26]. Unreduced gametes can be used to create new variants in breeding programs and have the unique advantages of transferring target genes that bypass self-incompatibility and stably transmitting heterozygosity [27]. Investigation of the mechanisms underlying the formation of natural doubling gametes in haploid peach can provide a theoretical basis for the use of natural doubling gametes in plant breeding.

Here, we demonstrated the presence of pollen grains with 2*x* (*x* means the basic number of chromosomes, and the number of chromosomes in a cell can be indicated by the multiple of *x*. In this paper, *x* and its multiple are used to represent the chromosome situation in gametophytes.) ploidy in the haploid ‘9-D’ by observing pollen grain morphology, diameters, analyses of viability, and meiotic chromosome behavior of pollen mother cells. The cytological mechanism behind the fertility trait of ‘9-D’ was discussed. Our results provide a theoretical basis for haploid breeding in fruit trees.

## Results

### Identification of ploidy

**Figure 1:**
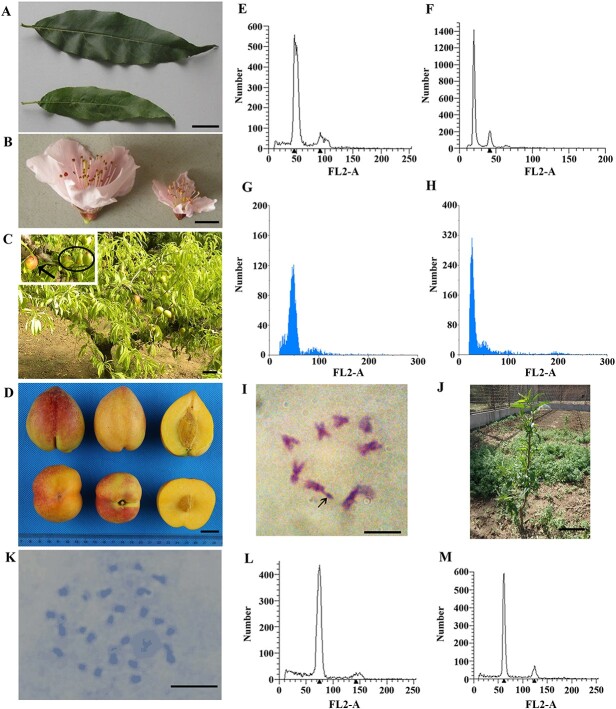
The morphological and cytological traits of ‘9-D’ and its offspring. **(A)** Comparison of ‘9-D’ and normal diploid leaves. The upper part is the diploid leaf and the lower part is the ‘9-D’ leaf. Bar = 2 cm. **(B)** Comparison of ‘9-D’ and normal diploid flowers. Diploid flowers on the left, ‘9-D’ flowers on the right. Bar = 1 cm. **(C)** Fruit state of ‘9-D’. The arrow points to the normally developing fruit, and in circles are those with abnormal development. Bar = 20 cm. **(D)** The fruit details of ‘9-D’. Bar = 2 cm. **(E)** Flow cytometry ploidy analysis of leaves of ‘Ruiguang 18 (RG18)’ (diploid). **(F)** Flow cytometry ploidy analysis of leaves of ‘9-D’. **(G)** Flow cytometry ploidy analysis of young flower buds of ‘RG18’ (diploid). **(H)** Flow cytometry ploidy analysis of young flower buds of ‘9-D’. **(I)** Chromosomes in ‘9-D’ somatic cells. Arrows point to the satellite. Bar = 100 μm. **(J)** Seeding offspring of ‘9-D’. Bar = 20 cm. **(K)** Chromosomes in the somatic cells of ‘9-D’ seeding offspring. Bar = 100 μm. **(L)** Flow cytometry ploidy analysis of young flower buds of ‘9-D’ seeding offspring (triploid) **(M)** Flow cytometry ploidy analysis of young flower buds of ‘9-D’ seeding offspring (triploid aneuploid).

Compared to normal diploid plants, ‘9-D’ was shorter; with smaller leaves, petals, and fruits; and weaker growth (Fig. 1a–d, Fig. S1). Thus, it was preliminarily deemed to be a haploid mutant. Later, flow cytometry ploidy analysis was conducted using the leaves and flower buds of ‘9-D’ along with its female parent ‘Ruiguang 18 (RG18)’, and the results further confirmed that ‘9-D’ is a pure haploid (Fig. 1e–h). Somatic tissues of ‘9-D’ were used to determine the number of chromosomes in the cells using the Giemsa staining method. The results showed that there were eight chromosomes in the cells (n = *x* = 8), and a satellite was observed on one chromosome (Fig. 1i). This cytological finding combined with the results of morphological identification as well as flow cytometry analysis demonstrated that ‘9-D’ is a pure haploid that contains a single set of chromosomes instead of a chimera. ‘9-D’ can produce offspring through self-pollination (Fig. 1j). Observation of the chromosomes in the somatic cells of the offspring, combined with flow cytometry analysis, showed that the offspring of ‘9-D’ were triploid or triploid aneuploid (Fig. 1k–m).

### Surface morphology of mature pollen grains

**Figure 2:**
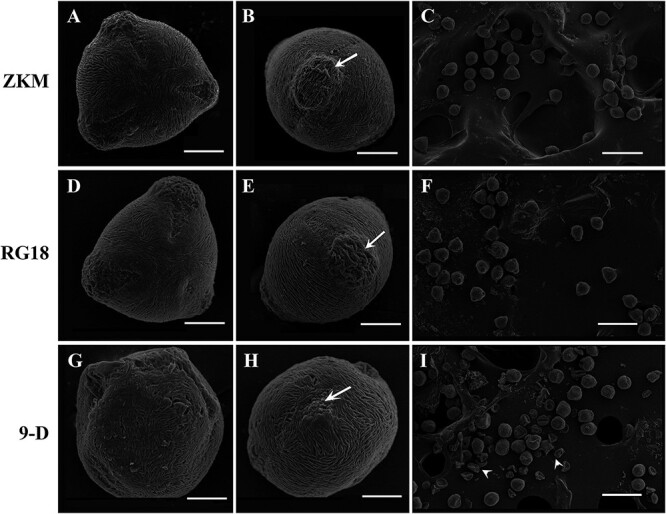
SEM micrographs showing the surface morphology of mature pollen grains of ‘ZKM’, ‘RG18’, and ‘9-D’. **(A–C)** ‘ZKM’. **(D–F)** ‘RG18’. **(G–I)** ‘9-D’. **(A, D, G)** Polar view of a single mature pollen grain. Compared with ‘ZKM’ and ‘RG18’, the pollen grains of ‘9-D’ showed an abnormal morphological structure. Bar = 10 μm. **(B, E, H)** The germinal aperture of a single mature pollen grain. Arrows point to the germinal aperture. Compared with ‘ZKM’ and ‘RG18’, the germinal aperture of ‘9-D’ could not protrude normally. Bar = 10 μm. **(C, F, I)** Local map of multiple mature pollen grains. Arrows point to abnormal pollen grains. Bar = 1 mm.

To investigate the cytological mechanism of fertile pollen formation in ‘9-D’, the structure of mature pollen grains of ‘9-D’ and its female parents was first investigated using a scanning electron microscope (SEM). It is well known that the normal pollen grains of peach species have a tricolporate structure. As a result, the mature pollen grains of ‘Zaokuimi (ZKM)’ and ‘RG18’ had normal pollen grain structures (Fig. 2a, d), while the pollen grains of ‘9-D’ showed irregular spherical shapes (Fig. 2g). The germinal aperture of ‘ZKM’ and ‘RG18’ developed normally and clearly (Fig. 2b, e). However, the pollen grains of ‘9-D’ had abnormal germinal aperture and could not protrude normally (Fig. 2h). The local map of multiple mature pollen grains showed that the majority of pollen grains of the parents were morphologically normal (Fig. 2c, f), whereas a considerable proportion of the haploid pollen grains were morphologically abnormal (Fig. 2i).

### Analysis of pollen viability and germination

**Figure 3:**
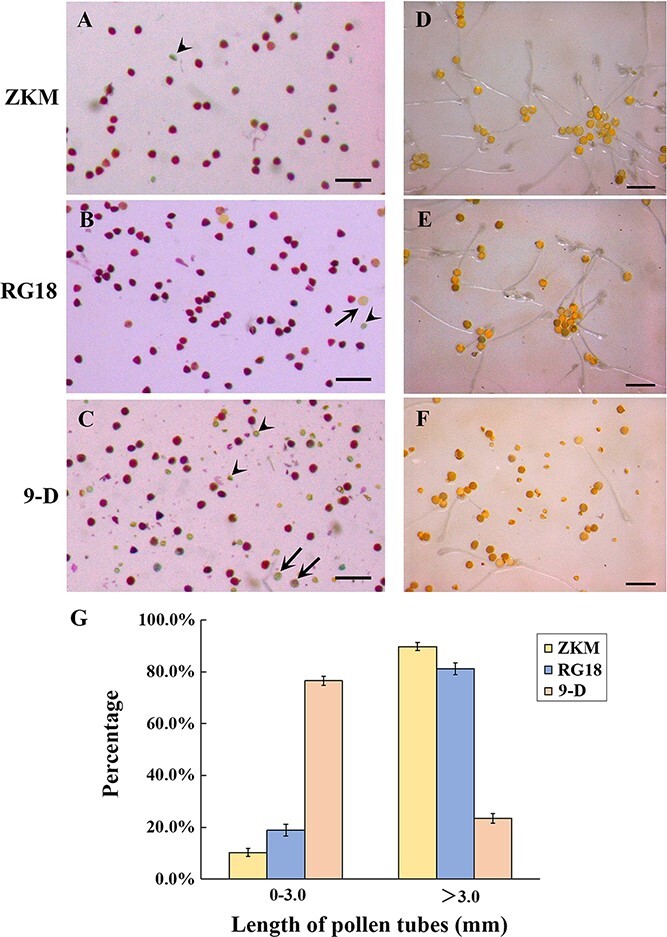
Pollen viability and germination rate of ‘ZKM’, ‘RG18’, and ‘9-D’. Alexander staining of mature pollen grains of **(A)** ‘ZKM’, **(B)** ‘RG18’, and **(C)** ‘9-D’. Arrow-shaped symbols point to normal but non-viable pollen grains. Arrow points indicate abnormal morphology of non-viable pollen grains. Bar = 2 mm. *In vitro* germination of mature pollen grains of **(D)** ‘ZKM’, **(E)** ‘RG18’, and **(F)** ‘9-D’. Bar = 2 mm. **(G)** Pollen tube length statistics for germination of mature pollen grains *in vitro* of ‘ZKM’, ‘RG18’, and ‘9-D’ (~50 counts per sample, repeated three times). The data are shown as mean ± standard deviation (SD) based on three biological replicates.

As mentioned above, SEM results showed a significant portion of pollen grains in ‘9-D’ that had abnormal development. Therefore, Alexander staining was further carried out to investigate the viability of pollen grains. This method can stain the cytoplasm of viable pollen grains purple color, but non-viable pollen grains were stained blue due to lack of the cytoplasm. The staining results showed that most of the mature pollen grains of ‘ZKM’ and ‘RG18’ were viable (Fig. 3a, b), and only a few were non-viable with normal or abnormal morphology (Fig. 3a, b). The mature pollen grains of haploid ‘9-D’ were predominantly non-viable with normal or abnormal morphology (Fig. 3c). Statistical analysis revealed that 1554 out of the 1669 pollen grains of ‘ZKM’ examined (93.1%) were viable, and 0.8% of all the examined pollen grains had abnormal pollen morphology. Of the 1850 pollen grains of ‘RG18’ examined, 1730 (93.5%) were viable and 20 (1.1%) had abnormal pollen morphology. Among the 2573 pollen grains of ‘9-D’ examined, 1297 (50.4%) were viable and 1148 (44.6%) had abnormal pollen morphology (Table S1).

Although the pollen viability of haploid ‘9-D’ was as high as 50.4%, its seed-setting percentage was very low. To gain insights into this abnormal phenomenon, we examined the germination rate of mature pollen grains *in vitro*. The results showed that most of the mature pollen grains of ‘ZKM’ and ‘RG18’ showed pollen tube elongation after 5 h of *in vitro* culture, and only a few pollen grains could not germinate (Fig. 3d, e). By contrast, only a few mature pollen grains of haploid ‘9-D’ could germinate normally (Fig. 3f). Statistical analysis showed that 51.5% of the 1872 pollen grains of ‘ZKM’ examined could germinate normally, and 8.4% of all the examined pollen grains had abnormal morphology. Among the 1918 pollen grains of ‘RG18’ examined, 53.8% could germinate normally and 4.3% had abnormal morphology. However, only 7.6% of the 2564 pollen grains of ‘9-D’ examined could germinate normally, and 38.5% had abnormal morphology (Table S2).

In addition, the growth length of the pollen tube was also estimated in this study. Statistical analysis showed that 10.3%, 18.9%, and 76.5% of pollen grains of ‘ZKM’, ‘RG18’, and ‘9-D’, respectively, had formed short pollen tubes with a length of 0–3 mm (Fig. 3g). Pollen grains of ‘ZKM’, ‘RG18’, and ‘9-D’ that had formed long pollen tubes with lengths >3 mm accounted for 89.7%, 81.1%, and 23.5%, respectively (Fig. 3g). The average pollen tube length was 5.09 mm for ‘ZKM’, 4.66 mm for ‘RG18’, and 2.69 mm for ‘9-D’ (Table S3). These results indicated that the pollen grains of haploid ‘9-D’ could germinate normally, but their growth was strongly inhibited.

### Chromosomal behavior of pollen mother cells at different stages of meiosis

**Figure 4:**
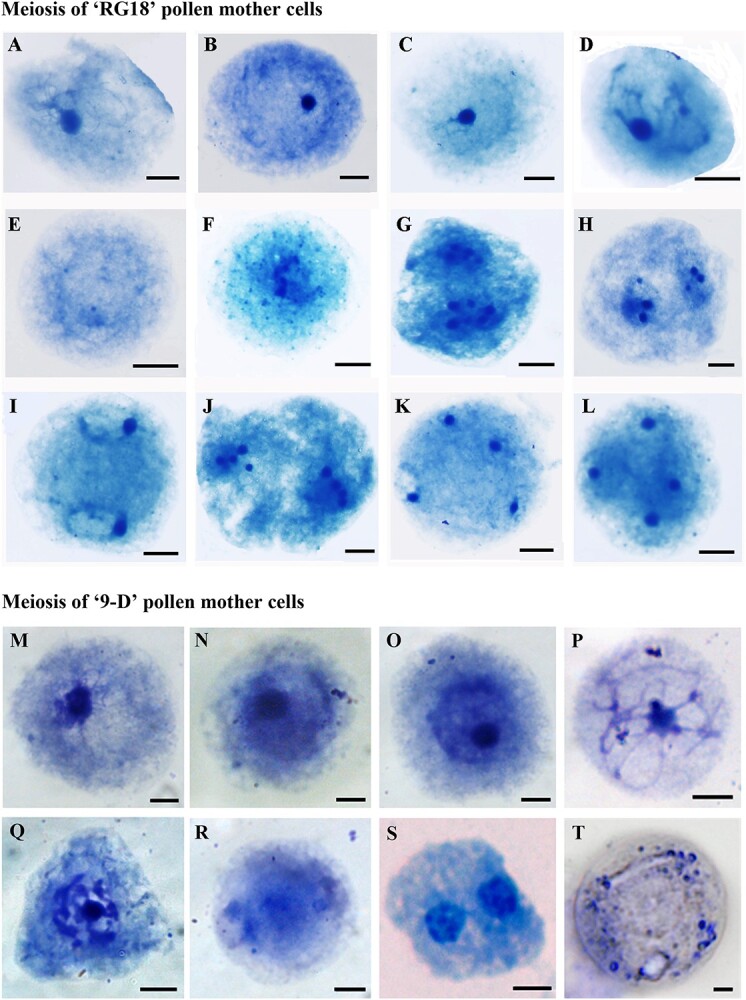
The meiosis of ‘RG18’ and ‘9-D’ pollen mother cells was observed by Giemsa staining. **(A)** Leptotene of ‘RG18’. **(B)** Zygotene of ‘RG18’. **(C)** Pachytene of ‘RG18’. **(D)** Diplotene of ‘RG18’. **(E)** Diakinesis of ‘RG18’. **(F)** Metaphase I of ‘RG18’. **(G)** Anaphase I of ‘RG18’. **(H)** Telophase I of ‘RG18’. **(I)** Prophase II of ‘RG18’. **(J)** Metaphase II of ‘RG18’. **(K)** Telophase II of ‘RG18’. **(L)** Tetrad of ‘RG18’. **(M)** Leptotene of ‘9-D’. **(N)** Zygotene of ‘9-D’. **(O)** Pachytene of ‘9-D’. **(P)** Diplotene of ‘9-D’. **(Q)** Diakinesis of ‘9-D’. **(R)** Metaphase I of ‘9-D’. **(S)** Metaphase II (or anaphase II) of ‘9-D’. **(T)** Telophase II of ‘9-D’. Bar = 10 μm.

Chromosome behaviors during the meiosis period were examined for ‘RG18’ and ‘9-D’ pollen mother cells using dewalled hypotonic Giemsa staining (Fig. 4, Fig. S4). The results indicated that the meiosis of pollen mother cells of ‘RG18’ was normal, while the meiosis of pollen mother cells of ‘9-D’ lacked a division phase between anaphase I and prophase II. Moreover, many phases were found at metaphase II or possibly anaphase II (Fig. 4S), and a lot of dyads were observed at the end.

**Figure 5:**
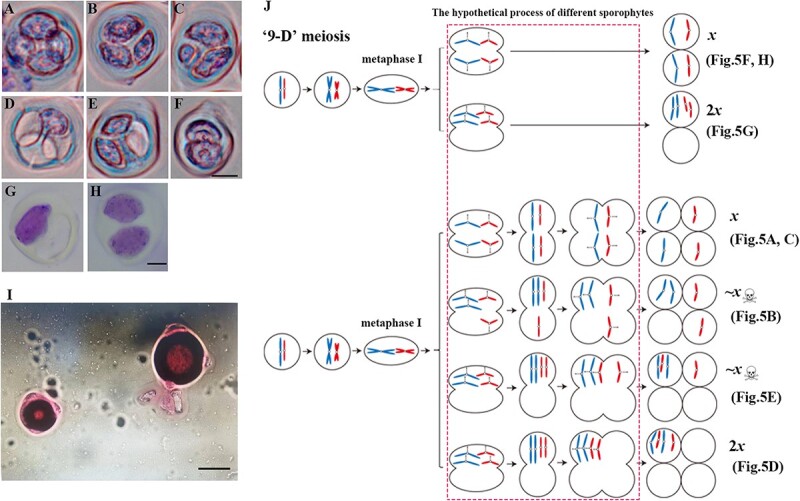
The tetrad stage of ‘9-D’ pollen mother cells was observed by Giemsa and Carbol-fuchsin staining, pollen grains of ‘9-D’ dyed by Alexander, and schematic summary of male gamete production by the ‘9-D’ pollen mother cell. **(A, C)** Normal tetrad. **(B)** Tetrad with three real and one empty. **(D)** Tetrad with one real and three empty. **(E)** Tetrad with two real and two empty. **(F, H)** Dyad with two real and zero empty. Bar = 10 μm. **(G)** Dyad with one real and one empty. **(I)** The larger one is a 2*x* ploidy pollen grain, and the smaller one is an *x* ploidy one. Bar = 10 μm. **(J)** In normal meiosis, chromosomes replicate once and separate twice. During the meiosis I, homologous chromosomes recombine and separate. During the meiosis II, sister chromatids separate. In ‘9-D’, the absence of homologous chromosomes and the unclear process of anaphase I to prophase II lead to the production of various balanced or unbalanced gametes. This schematic shows the possible processes by which various male gametes are produced.

Since abnormalities were detected in the tetrad stage of ‘9-D’ pollen mother cells, the carbol-fuchsin staining experiment was conducted for pollen mother cells at this stage. The results revealed various types of sporophyte morphologies, such as anomalous dyads and anomalous tetrads (Fig. 5a–h, Fig. S5). Statistical analysis showed that 113 (44.49%) out of the examined 254 spores were dyads morphs (2 real) and 39.37% were normal tetrads morphs (4 real, 0 empty). These two forms accounted for the vast majority of sporophytes (Table 1). This is different from the meiosis process observed in normal diploid peach mother cells, with the presence of both one real and one empty in dyads (1.58%) as well as one real and three empty in tetrads (0.39%), which could theoretically produce gametes with 2*x* ploidy. A further possibility exists that these produce gametes with *x* ploidy that undergo a replication at the stage of microspore, thus becoming 2*x* pollen grains. Both these *x* and 2*x* gametes may eventually form fertile pollen, which is likely responsible for the self-fruitful ability of ‘9-D’. We summarize schematically the production of dyads morphs and tetrads morphs by meiosis in ‘9-D’ and the ploidy of their gametes (Fig. 5j).

### Existence of pollen with 2x ploidy in ‘9-D’

It is generally believed that there is a correlation between pollen diameter and its ploidy. If the diameter of a pollen grain increases by 30%, its ploidy level will double [28]. Thus, the diameter of the pollen of ‘9-D’ was determined (Fig. 5i) and the number of pollen grains of *x* and 2*x* was counted (Table 2). As a result, the *x* pollen was found to be the predominant type in ‘9-D’, accounting for 95.5% of all tested pollen grains. A small number of 2*x* pollen was also found, accounting for only 4.5% of all tested pollen grains (Table 2).

## Discussion

The frequency of haploid occurrence in peach under nature conditions is only 0%–0.7%, which is extremely rare, thus the haploids are excellent germplasm with great value in the fields of genetics, plant breeding, physiology, and embryology [5, 29, 30]. ‘9-D’ is a haploid derived from a cross-breeding program, planted in a greenhouse with a controlled growing environment. The flowering period of ‘9-D’ did not coincide with other varieties, and its self-breeding offspring are mostly triploid (data unpublished). Over the years, we have used ‘9-D’ as the male parent to cross with other diploid peaches and related species of peach, such as *Prunus sibirica* L., *Prunus tangutica* (Batalin) Koehne, *Prunus armeniaca* L., *Prunus triloba* Lindl., *Prunus davidiana* (Carrière) Franch., etc. Only the hybrid combination of *P. sibirica* × ‘9-D’ harvested 77 plants, and related research on progeny is ongoing. Since peach has a high heterozygosity genome, it is not possible to verify whether ‘9-D’ is derived from the female parent or male parent by markers. Currently, we are mining the single nucleotide polymorphism (SNP)  through next-generation sequencing data, and by analyzing the number of suspected SNP between parents, offspring and non-offspring, we have tentatively confirmed that ‘9-D’ is from the female parent ‘RG18’ (Table S4). Monet and Bassi [3] reported that the offspring of peach haploid are triploid, diploid, or aneuploid. However, there are no reports on the mechanisms underlying the fertility of peach haploids. In this study, we report for the first time the cytological mechanism of fertility in the peach haploid ‘9-D’ that produces triploid offspring.

**Figure 6:**
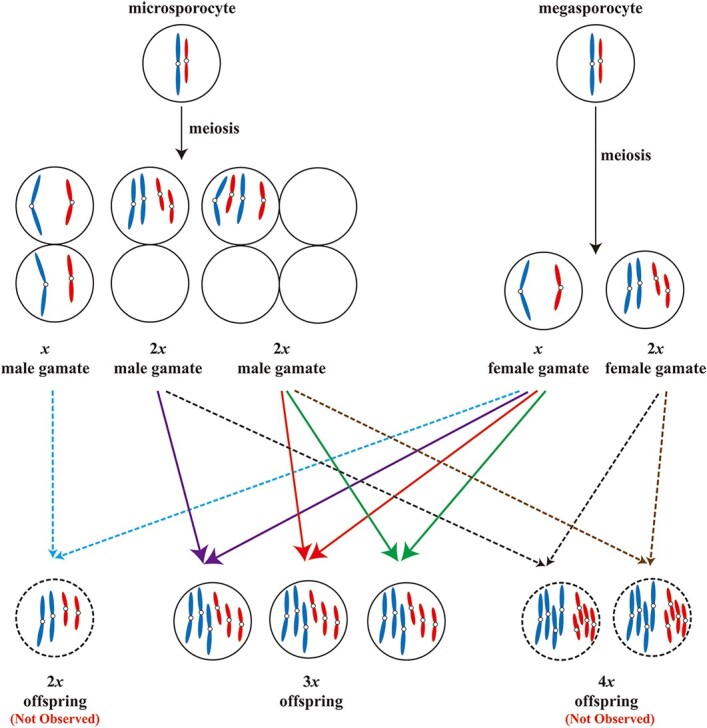
Schematic summary of offspring produced by peach haploid ‘9-D’

Pollen viability and germination analysis showed that the pollen grain growth of ‘9-D’ was strongly inhibited. As a result, even if these poorly developed pollen grains could be bound to the stigma, pollen tubes could not grow normally to finish fertilization. Thus, pseudo-fertilization may occur, which causes a termination of fruit development at certain stages or the formation of seedless fruits. This hypothesis is consistent with the finding that the extremely low rate of seed setting in the haploid ‘9-D’. Since the number of female gametophytes is limited and the morphological image of megaspore is difficult to be captured, the occurrence of microspore with 2*x* ploidy in haploid ‘9-D’ was first observed. According to the correlation between pollen diameter and ploidy level [28], the proportion of 2*x* pollen in ‘9-D’ was estimated to be ~4.5%. This result is basically equivalent to the seed-setting rate of ‘9-D’. Given the fact that ‘9-D’ has a very low rate of seed setting and the majority of fruits have no embryos due the cessation of fruit development after a period of time, we speculate it is more likely that the 2*x* male gametophyte fuses with a *x* female gametophyte to develop 3*x* offspring (Fig. 6). Although the occurrence of megaspore with 2*x* ploidy in ‘9-D’ remains uncertain, the possibility cannot be excluded that the *x* male gametophyte fuses with a 2*x* female gametophytes to generate 3*x* offspring (Fig. 6). However, the number of female gametophytes is far less than that of male gametophytes, and thus the 3*x* offspring derived from the 2*x* female gametophytes are extremely rare or negligible. Since only triploid and triploid aneuploidy were observed in the offspring of ‘9-D’, it cannot be excluded that the fertility may be partially due to fusion of the *x* male gametophyte with the *x* female gametophyte that eventually produces 2*x* offspring, and 2*x* male gametophyte with the 2*x* female gametophyte that eventually produces 4*x* offspring (Fig. 6). Up till now, most of the studies on haploid germ cytology use the male gametophyte (microspore) as the research material, and few reports are available on the female gametophyte. In the future, more studies are still needed to comprehensively uncover the mechanism of the fertility in ‘9-D’.

Meiosis is a kind of conserved cell division, which is very important for the sexual reproduction of eukaryotes. However, under the influence of internal and external factors, plant sporophytes can occasionally produce unreduced gametes during meiosis [31, 32]. In haploids, unreduced gametes can restore fertility. Drones are typical representatives of haploids that restore fertility by producing unreduced gametes. Drones develop from unfertilized egg cells. The spermatocyte performs a special form of meiosis. The key to this special meiosis is the failure of the cytoplasmic division at the end of the first meiotic division. The chromosomes are pulled toward one pole, thus avoiding the equal distribution of chromosomes in the cell to retain the fertility of the male gametophyte. The resulting male gametophyte has the same ploidy as the spermatocyte. This particular meiosis is called ‘pseudomeiosis’ [33]. A similar pattern of division has been found in plants. In *Arabidopsis thaliana osd1* mutants, the meiosis process lacked meiosis II, resulting in a large number of dyads (unreduced gametes), which produced tetraploid plants after self-mating [34, 35]. In addition, the researchers constructed the *osd1/AtRec8/ATSPOL-1* triple mutant, in which the meiosis I is completely replaced by a mitotic and the meiosis II is lost. This phenotype is called the *MiMe* ([35], 2009). Later, the *MiMe* phenotype was successfully introduced in rice [36]. *MiMe* produces unreduced gametes that are identical with the maternal genetic composition and is a key step in the artificial creation of apomixes. Interestingly, our results showed that the meiosis process in ‘9-D’ resulted in a significant proportion of dyads, similar to the phenotype reported in *A. thaliana osd1* mutants. However, haploid ‘9-D’ seems to produce 2*x* gametes, which may be due to the fact that the ‘9-D’ pollen mother cell lacked the anaphase I to prophase II process during meiotic, making it more likely that chromosomes can be pulled to one side. Thus, both homologous chromosomes and sister chromatid separation could be affected, leading to male gamete chromosome doubling. We speculate that the fertility of ‘9-D’ is related to the formation of 2*x* gametes, and this other mechanism of pseudomeiosis deserves further investigation.

Haploid and its naturally doubled 2*x* gametes have important value in genetic studies and plant breeding. Haploid can produce double haploid by chromosome doubling. A double haploid population is very useful for genetic linkage map construction and QTL mapping, which is an important object of genomics research [37, 38]. The double haploid population can allow the accurate detection of candidate genes, thus laying a foundation for plant breeding [39]. The production of naturally doubled gametes plays a very important role in the sexual polyploidy of plants [40]. Studies have shown that naturally doubled gametes are involved in the production of polyploidy of many plants, such as *Solanum* and *Rosa*, which is one of the ways to give rise to new plant species [41–44]. Polyploidy can enhance the adaptability and competitiveness of plants. Understanding the mechanism of naturally doubled gamete formation will facilitate the use of naturally doubled gametes in plant breeding programs [40, 45, 46].

**Table 1:** The number of various types of sporophytes in the tetrad stage of ‘9-D’ pollen mother cells

Sporophytes type	Sporophytic characteristics	Percentage
Undivided sporophyte		0.79%
Dyad	2 real, 0 empty	44.49%
	1 real, 1 empty	1.58%
	0 real, 2 empty	0.79%
Tetrad	Normal tetrad	39.37%
	2 real, 2 empty	3.94%
	3 real, 1 empty	8.65%
	1 real, 3 empty	0.39%

**Table 2:** Diameter and proportion of pollen grains with different ploidy of ‘9-D’

Ploidy type	Diameter of pollen grains (μm)	Number	Proportion (%)
*x*	40.24 ± 4.16	191	95.5
2*x*	61.56 ± 7.09	9	4.5

At present, poplar is the model plant of forest trees, but it cannot be used to investigate fruit-related traits. Peach is a woody and self-pollinated plant species, with a relatively simple genetic background and small chromosome number. Its genome is small (~227.4 Mb), about twice that of *A. thaliana* and only half that of poplar [47]. It has a short juvenile period of 2–3 years and is a model plant for the genetic studies of perennial fruit trees [48]. Therefore, it is worthy of further studies on fertility of haploid in peach in the future.

## Conclusions

This study investigated the cytological mechanism underlying the fertility in the haploid ‘9-D’ by examining the chromosomal behavior of pollen mother cells at different stages of meiosis and the morphological characteristics of the pollen. In ‘9-D’, 50.4% of pollen grains were viable. The ‘9-D’ pollen mother cells lacked the anaphase I to prophase II process during the meiotic. 2*x* pollen did exist in ‘9-D’ and it fused with the *x* female gametophyte to produce the 3*x* offspring. This is probably the reason why the haploid ‘9-D’ recovered partial fertility. The breeding process of peach ploidy is relatively backward due to the lack of germplasm resources, the imperfect technical system of ploidy breeding, and the insufficient depth of related theoretical research. This study can provide the theoretical basis for the ploidy breeding and genetic analysis in peach.

## Materials and methods

### Plant material

The haploid ‘9-D’ (*P. persica* ‘9-D’) as well as its female parent ‘Ruiguang 18 (RG18)’ (*P persica* ‘RG18’) (Fig. S2, Table S5) and male parent ‘Zaokuimi (ZKM)’ (*P persica* ‘ZKM’) (Fig. S3, Table S5) used in this study are maintained in the demonstration base of Institute of Forestry and Pomology, Beijing Academy of Agriculture and Forestry Sciences, Haidian District, Beijing, China.

### Flow cytometry

The fresh leaves (0.1 g) of the plants were taken and chopped with a sharp blade in 2 ml of cell lysis buffer. The filtrate was filtered and collected through a filter membrane with a pore size of 80 μm, centrifuged at 4°C at 1000 rpm for 3 min. The supernatant was discarded and the precipitated cells were collected. The nuclear DNA was fluorescentially labeled with Propidium iodide (PI) solution or DAPI staining solution, and the samples were stained in the dark for 20 min. Then the ploidy of the samples was identified by FACSCalibur flow cytometry. Samples with known ploidy were used as control samples, and the particles were completely displayed on the FSC/SSC chart by adjusting the voltage parameters of FSC and SSC. The horizontal coordinate of the control sample was fixed by adjusting the voltage parameter of FL2, and the above parameters were saved. The test sample was detected, and the ploidy of the test sample was determined according to the peak location and reference to the peak location of the control sample.

### Scanning electron microscope observation

Mature anthers of ‘ZKM’, ‘RG18’, and ‘9-D’ were collected and placed into 1.5-ml centrifuge, tubes respectively. Then, the prepared fixation solution (3% glutaraldehyde and 4% paraformaldehyde) was added into the tubes and fixed at 4°C overnight. Rinse with phosphate buffer (0.1 mol/l, pH 7.2) three times, 1 h each time. Later the samples were dehydrated in ethanol series (one time in 30%, 50%, 70%, 90%, and 100%, 20 min each). Finally, the samples were dried naturally, coated gold, and observed under the SEM.

### Pollen grain viability estimated

Dropped the Alexander staining solution onto the microscope slide, picked up the anther with tweezers, and shook the pollen onto the Alexander staining solution. Then covered the coverslip and waited for 10–15 min [49]. Finally, placed the microscope slides under the microscope for observation (OLYMPUS SZX7, OLYMPUS DP73).

### Pollen Germinability determination

The pollen medium was cut into small pieces of ~0.5 cm^2^ and placed on a slide, which was then placed in a wet box. Pollen were gently applied to the pollen medium and only one flower was applied to each slice. The boxes containing pollen were placed in a culture environment at 23°C for 5 h, photographed, and counted under a microscope (OLYMPUS SZX7, OLYMPUS DP73).

### Giemsa staining

The buds in the stage of meiosis were collected and fixed overnight with a Carnoy’s fixative, which is composed of methyl alcohol and acetic acid (3:1). The anthers were removed from the fixed buds and soaked for >20 min after being washed twice in distilled water. The cleaned anthers were cut and placed into 1.5-ml centrifuge tubes, with four anthers per tube. Added 300 μl of distilled water to the centrifuge tubes to free the pollen mother cells into the distilled water. The sample was centrifuged at 2000 rpm for 5 min and the supernatant was removed. Added 300 μl of mixed enzyme solution (3% cellulase, 0.3% pectinase, and 1% Snailase) and enzymolysis at 37°C for 2.5 h. Added 300 μl of distilled water, shook, and centrifuged at 1000 rpm for 3 min, removed the supernatant. Added 200 μl of fixing solution and fixed for 5 min. Absorbed 10 μl of the mixture onto the slides, and when there was little fixing solution left on the surface of the slides, baked the material evenly with an alcohol lamp for several seconds to fix the material. Dyed with 5% of Giemsa staining solution for 10 min and examined under a microscope (OLYMPUS BX51, OLYMPUS DP73).

### Carbol-fuchsin staining

During the flower bud meiosis, samples were collected every 3 h once a day. The taken buds were immediately placed in the newly prepared Carnoy’s fixative at 4°C for 24 h. If the materials need to be preserved, they can be directly changed into 70% alcohol and stored in the refrigerator. The anthers were taken out and dissociated with 1 mol/l hydrochloric acid solution at 60°C for 5–6 min, and then soaked in distilled water for 5 min. The samples were stained with modified Carbol-fuchsin for 5–8 min and photographed under a microscope (OLYMPUS BX51, OLYMPUS DP73).

## Supplementary Material

Web_Material_uhae316
