# Supplementary material for: The cytological mechanism of the peach haploid producing triploid offspring
Source: Hortic Res. 2024 Nov 18;12(2):uhae316. doi: 10.1093/hr/uhae316 (PMC11817870; doi:10.1093/hr/uhae316)
Supplement: Web_Material_uhae316 [file web_material_uhae316.zip › Revised Supplementary Figure 1-5.docx]

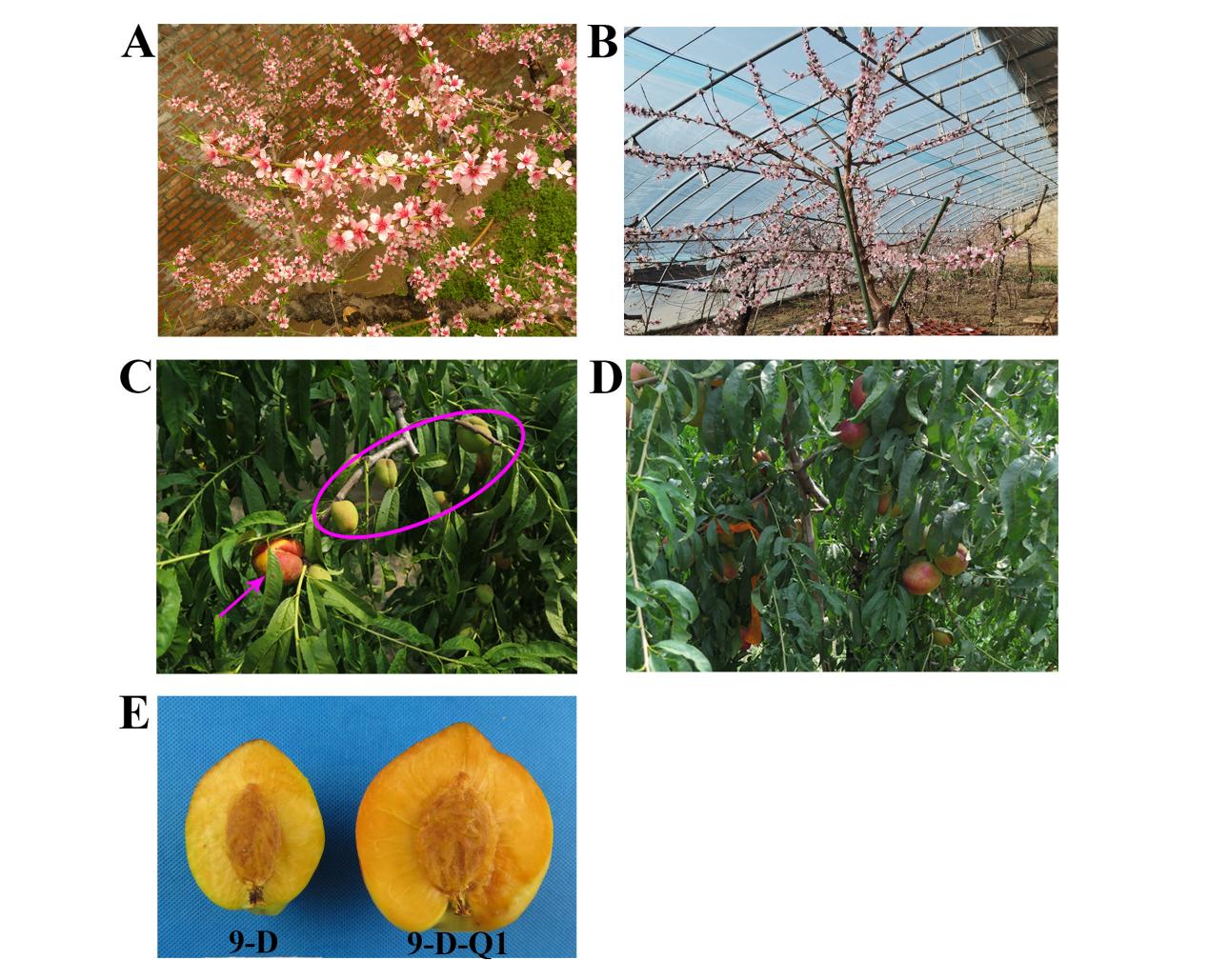


**Figure S1**. Flowering status and fruit characters of ‘9-D’ and its doubled chimera‘9-D-Q1’. **(A)** Flowering status of ‘9-D’. **(B)** Flowering status of ‘9-D-Q1’. **(C)** Fruit ripening state of ‘9-D’. The arrow points to the fruit that is normally developing and ripe, and in circles are those with abnormal development. **(D)** Fruit ripening state of ‘9-D-Q1’. **(E)** Fruit comparison of ‘9-D’ and ‘9-D-Q1’.


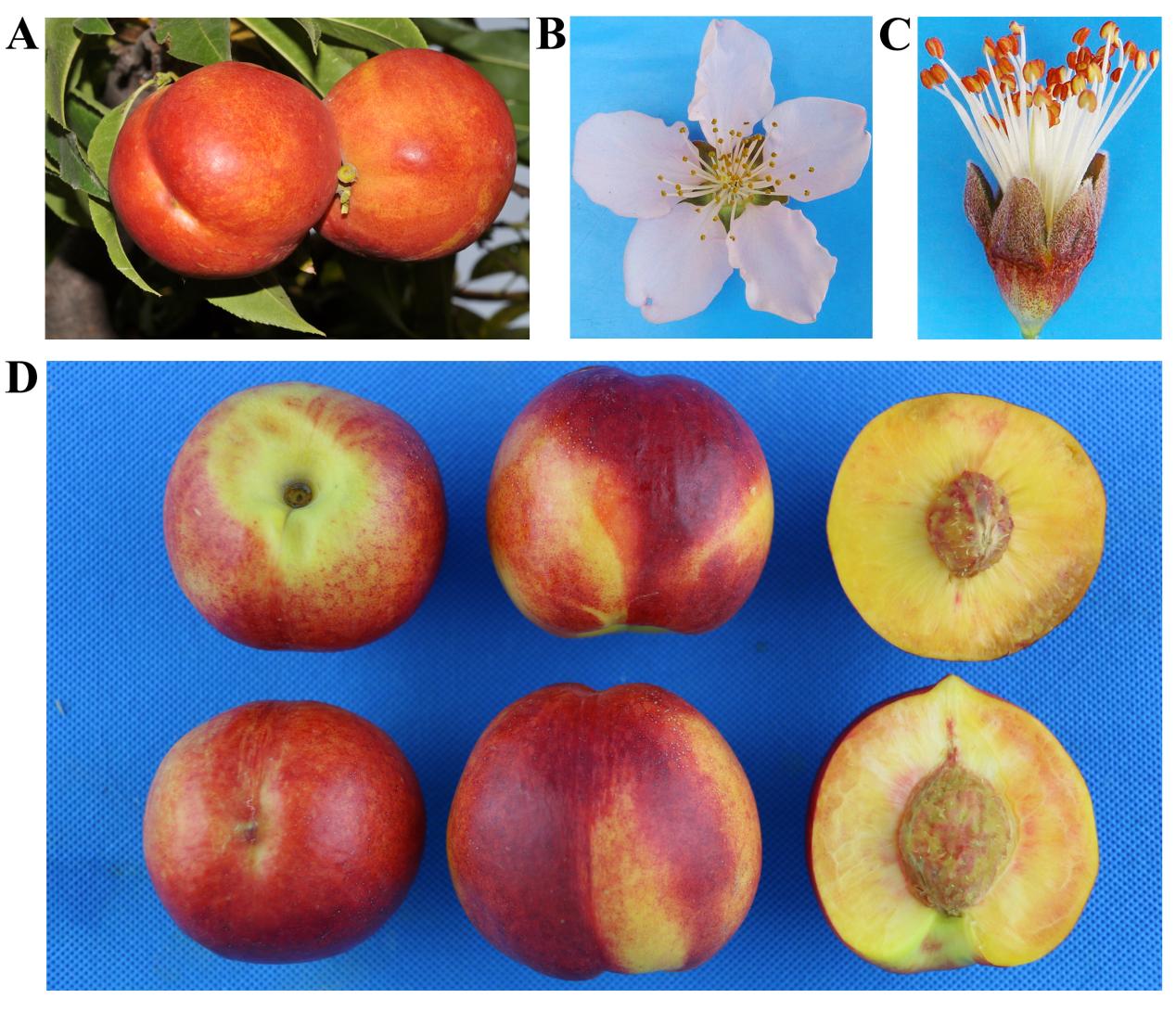


**Figure S2**. Standard photo of ‘RG18’.**(A)** Fruiting status. **(B)** Corolla **(C)** Stigma **(D)** Fruit.


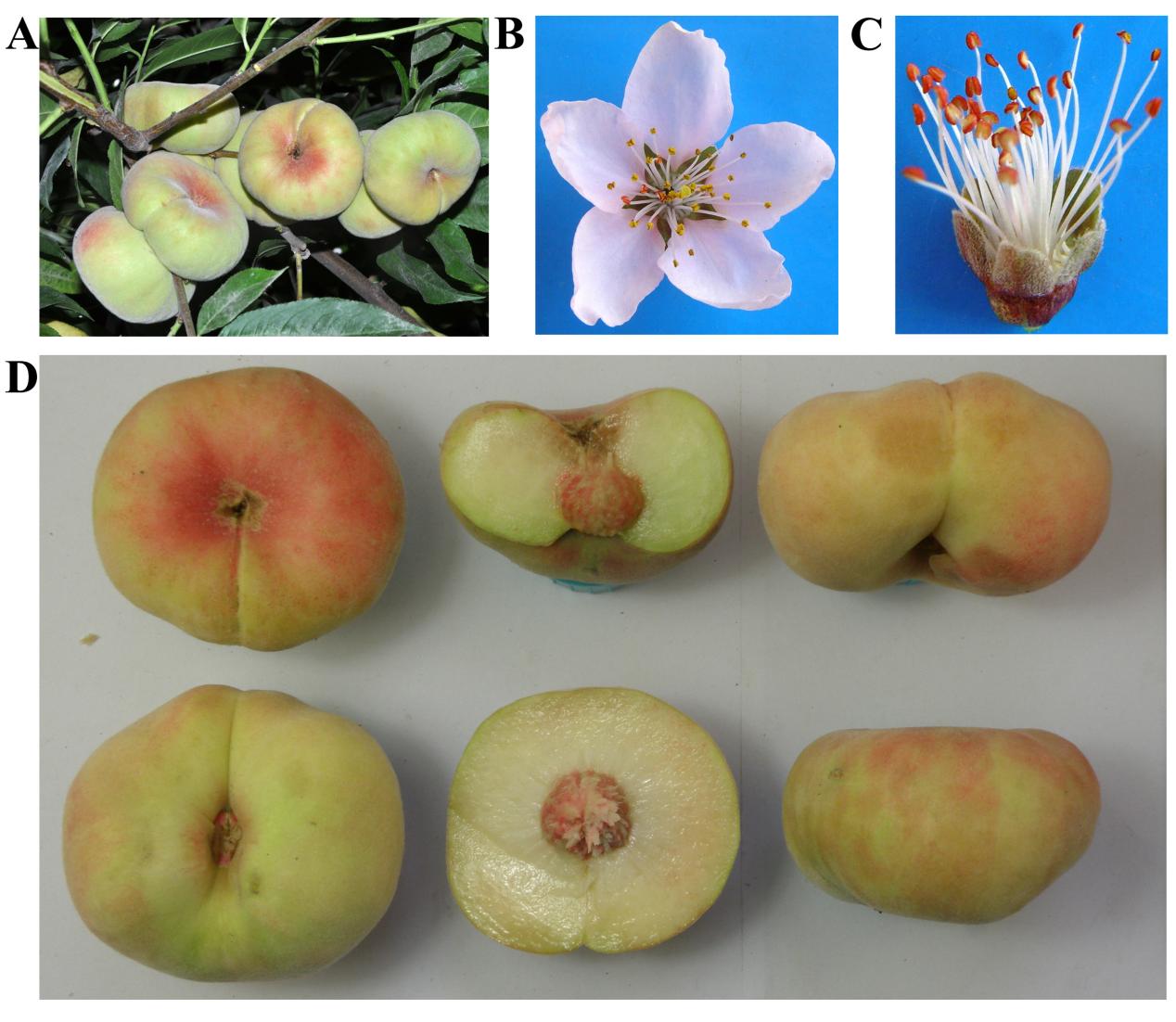


**Figure S3**. Standard photo of ‘ZKM’. **(A)** Fruiting status. **(B)** Corolla **(C)** Stigma **(D)** Fruit.


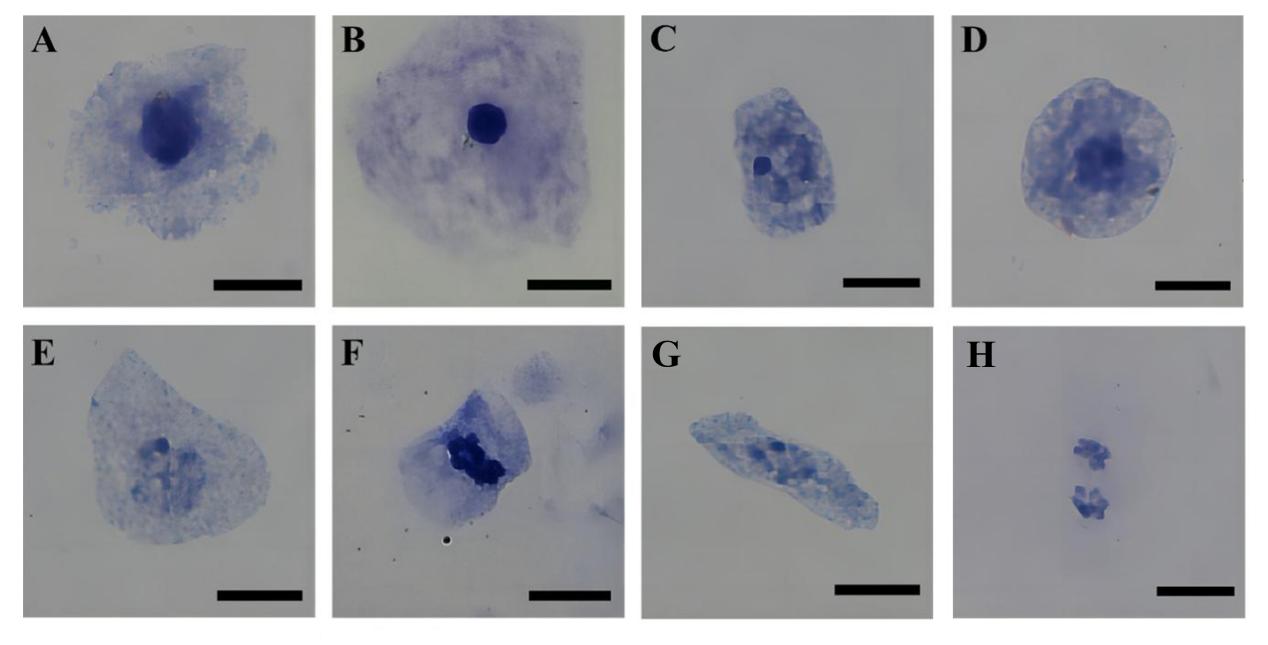


**Figure S4**. The meiosis of ‘9-D’ pollen mother cells was observed by Giemsa staining. **(A)** Leptotene **(B)** Zygotene **(C)** Pachytene **(D)** Diplotene **(E)** Diakinesis **(F)** Metaphase I **(G, H)** Anaphase II. Bar = 10 µm.


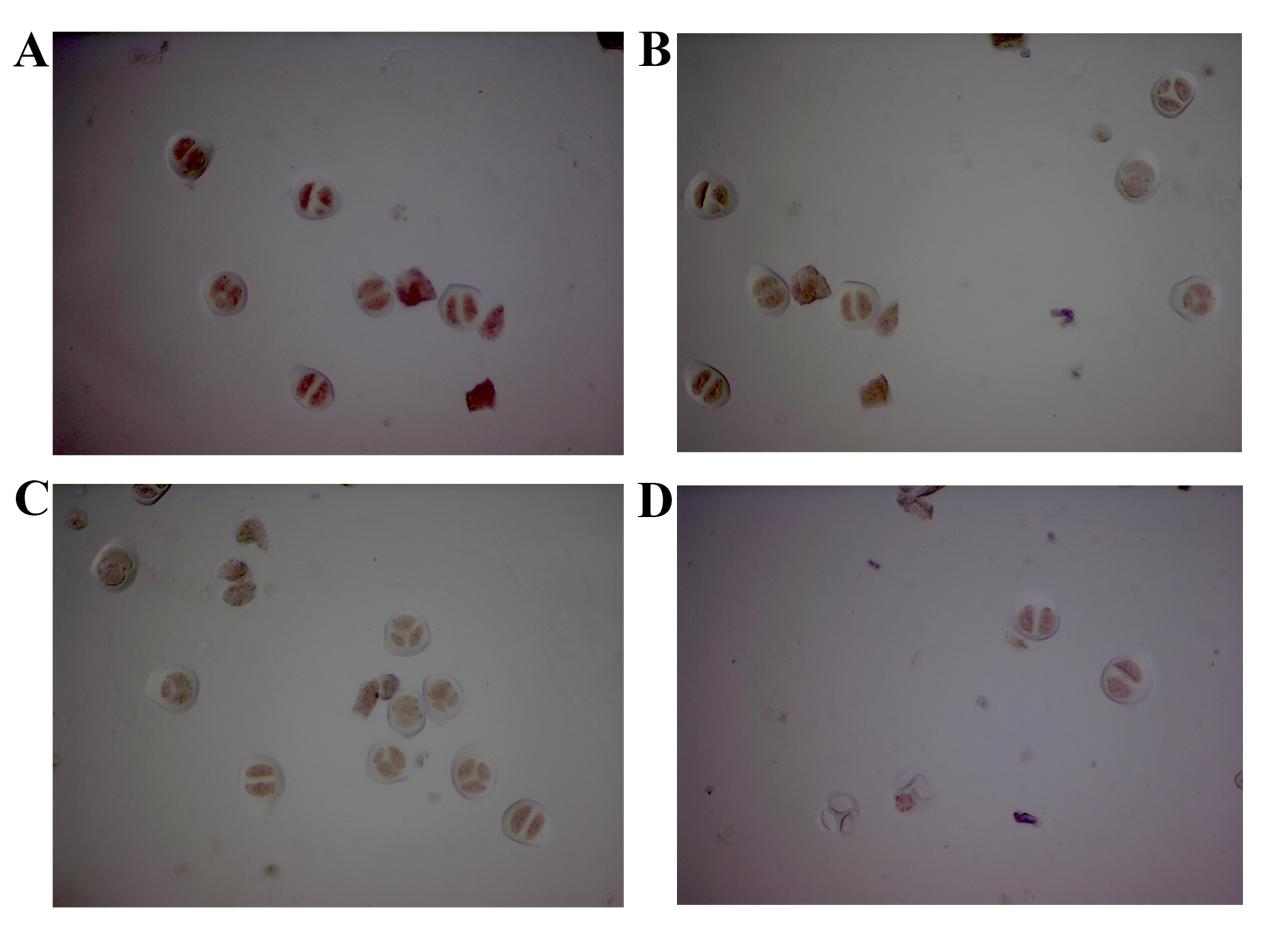


**Figure S5**. The tetrad stage of ‘9-D’ pollen mother cells were observed by aceto-carmine staining.
