# Supplementary material for: The cytological mechanism of the peach haploid producing triploid offspring
Source: Hortic Res. 2024 Nov 18;12(2):uhae316. doi: 10.1093/hr/uhae316 (PMC11817870; doi:10.1093/hr/uhae316)
Supplement: Web_Material_uhae316 [file web_material_uhae316.zip › Revised Supplementary Table 1-5-For reviewers.docx]

**Table S1.** Mature pollen grains viability statistics of ‘ZKM’, ‘RG18’ and ‘9-D’.

|  | ZKM | RG18 | 9-D |
| --- | --- | --- | --- |
| Percentage of viable pollen | 93.1% | 93.5% | 50.4% |
| Percentage of non-viable pollen (normal morphology) | 6.1% | 5.4% | 5.0% |
| Percentage of non-viable pollen (morphological abnormalities) | 0.8% | 1.1% | 44.6% |

**Table S2.** The germination rate of mature pollen grains of ‘ZKM’, ‘RG18’ and ‘9-D’.

|  | ZKM | RG18 | 9-D |
| --- | --- | --- | --- |
| Percentage of pollen germination | 51.5% | 53.8% | 7.6% |
| Percentage of pollen non-germination (normal morphology) | 40.1% | 41.9% | 53.9% |
| Percentage of pollen non-germination (morphological abnormalities) | 8.4% | 4.3% | 38.5% |

**Table S3.** Germination statistics of mature pollen grains of ‘ZKM’, ‘RG18’ and ‘9-D’.

|  | ZKM | RG18 | 9-D |
| --- | --- | --- | --- |
| Length of pollen tube (mm) | 5.09±0.48 | 4.66±0.64 | 2.69±0.53 |

**Table S4.** SNP types of different samples, and the suspected SNP of offspring.

1. With ‘RG18’ as parents and ‘9-D’/‘D’ as offspring, the suspected SNP was marked.

| Genotype | | RG18 | | | Suspected SNP/Total Stat SNP(%) |
| --- | --- | --- | --- | --- | --- |
|  |  | 0/0 | 0/1 | 1/1 |  |
| 1. D   (Offspring of ‘RG18’) | 0/0 | 179,514 | 458,641 | 144 ^a^ | 0.0271 |
|  | 1/1 | 29 ^a^ | 96 | 58 |  |
| D  (Unrelated haploid) | 0/0 | 879 | 241,733 | 116 ^a^ | 20.009 |
|  | 1/1 | 103,108 ^a^ | 169,934 | 112 |  |

a Assuming that ‘RG18’ is the parent of ‘9-D’/‘D’, the number marked in red is the number of suspected SNP of the offspring.

1. With ‘Legrand’ as parents and ‘RG18’/‘Zhong You 9’ as offspring, the suspected SNP was marked.

| Genotype | | Legrand | | | Suspected SNP/Total Stat SNP(%) |
| --- | --- | --- | --- | --- | --- |
|  |  | 0/0 | 0/1 | 1/1 |  |
| RG18  (Offspring of ‘Legrand’) | 0/0 | 71,212 | 10,719 | 7,631 ^b^ | 21.03 |
|  | 1/1 | 16,321 ^b^ | 2,876 | 5,130 |  |
| Zhong You 9  (Offspring of ‘Hong Shan Hu’) | 0/0 | 69,787 | 9,833 | 9,447 ^b^ | 29.59 |
|  | 1/1 | 27,408 ^b^ | 2,831 | 5,262 |  |

b Assuming that ‘Legrand’ is the parent of ‘RG18’/‘Zhong You 9’, the number marked in red is the number of suspected SNP of the offspring.

1. With ‘Hong Shan Hu’ as parents and ‘RG18’/‘Zhong You 9’ as offspring, the suspected SNP was marked.

| Genotype | | Hong Shan Hu | | | Suspected SNP/Total Stat SNP(%) |
| --- | --- | --- | --- | --- | --- |
|  |  | 0/0 | 0/1 | 1/1 |  |
| Zhong You 9  (Offspring of ‘Hong Shan Hu’) | 0/0 | 59,044 | 44,009 | 3,961 ^c^ | 6.26 |
|  | 1/1 | 5,332 ^c^ | 21,360 | 14,763 |  |
| RG18  (Offspring of ‘Legrand’) | 0/0 | 41,211 | 60,103 | 5,131 ^c^ | 12.94 |
|  | 1/1 | 12,547 ^c^ | 9,163 | 8,501 |  |

c Assuming that ‘Hong Shan Hu’ is the parent of ‘RG18’/‘Zhong You 9’, the number marked in red is the number of suspected SNP of the offspring.

**Table S5.** Fruit trait of ‘ZKM’, ‘RG18’ and ‘9-D’.

| Fruit Trait | 9-D | RG18 | ZKM |
| --- | --- | --- | --- |
| Type | Nectarine | Nectarine | Flat-peach |
| Shape | Round | Round | Flat |
| Weight (g) | 96 | 210 | 130 |
| Shape of pistil end | Pointed | Flat | Depressed |
| Symmetry | Slightly-asymmetric | Slightly-asymmetric | Slightly-asymmetric |
| Prominence of suture | Weak | Weak | Medium |
| Depth of stalk cavity | Deep | Medium | Shallow |
| Width of stalk cavity | Medium | Medium | Broad |
| Ground color | Yellow | Yellow | Greenish-white |
| Over colour | Medium-red | Dark-red | Light-red |
| Proportion of over color | Large | Very-large | Medium |
| Pubescence | Absent | Absent | Present |
| Flesh colour | Yellow | Yellow | White |
| Flesh texture | Firm-melting | Firm-melting | Soft-melting |
| Sweetness (soluble solids content in ° Brix) | 12-16 | 9-12 | 12-15 |
| Acidity | Low | Low | Low |
| Adhesion | Clingstone | Clingstone | Freestone |
| Fruit developing period (d) | 120 | 104 | 70 |
